# Botulinum Toxin a Valuable Prophylactic Agent for Migraines and a Possible Future Option for the Prevention of Hormonal Variations-Triggered Migraines

**DOI:** 10.3390/toxins11080465

**Published:** 2019-08-08

**Authors:** Lorena Dima, Andreea Bălan, Marius Alexandru Moga, Cătălina Georgeta Dinu, Oana Gabriela Dimienescu, Ioana Varga, Andrea Elena Neculau

**Affiliations:** 1 Department of Fundamental Disciplines and Clinical Prevention, Faculty of Medicine, Transilvania University of Brasov, 500019 Brasov, Romania; 2 Department of Medical and Surgical Specialties, Faculty of Medicine, Transilvania University of Brasov, 500019 Brasov, Romania; 3 Department of Law, Faculty of Law, Transilvania University of Brasov, 500019 Brasov, Romania

**Keywords:** Botulinum toxin type A, premenstrual migraine, chronic migraine, menopausal transition, neuropathic pain

## Abstract

Background: In 1989, Botulinum toxin (BoNT) was accepted by the FDA for the management of some ophthalmic disorders. Although it was initially considered a lethal toxin, in recent times, Botulinum toxin A (BoNT-A), which is the more used serotype, has expanded to cover different clinical conditions, primarily characterized by neuropathic pain, including migraines and headaches. Evidence suggests that migraines are influenced by hormonal factors, particularly by estrogen levels, but very few studies have investigated the prevalence and management strategies for migraines according to the hormonal status. The effects of several therapeutic regimens on migraines have been investigated, but the medications used varied widely in proven efficacies and mechanisms of action. BoNT-A is increasingly used in the management of migraine and several placebo-controlled trials of episodic and chronic migraine are currently underway. This paper is a review of the recently published data concerning the administration of BoNT-A in the prevention of chronic migraines. Considering the lack of population-based studies about the effectiveness of BoNT-A in the alleviation of premenstrual and perimenopausal migraines, this study proposes a new perspective of the therapeutic approach of migraine syndrome associated with menopausal transition and the premenstrual period. Methods: We selected the reviewed papers from CrossRef, PubMed, Medline, and GoogleScholar, and a total of 21 studies met our inclusion criteria. Results: To date, no specific preventive measures have been recommended for menopausal women with migraines. BoNT-A often reduces the frequency and intensity of migraine attacks per month; the treatment is well tolerated and does not exhibit a significantly higher rate of treatment-related side effects. No population-based studies were conducted in order to highlight the role of BoNT-A in menopause-related migraines, neither in menstrual migraines. Conclusion: There is a need for further research in order to quantify the real burden of menstrual and perimenopausal migraines and to clarify if BoNT-A could be used in the treatment of refractory postmenopausal and premenstrual migraines.

## 1. Introduction

Migraines are a disabling episodic brain disorder, which is more common in women than in men and is frequently undertreated [1]. A study on US population reported higher incidence of migraines in women (18.2%) compared with men (6.5%), with nearly 23% of families with one family member suffering from it. Some differences imposed by ethnicity and race were also observed, with migraine prevalence being higher in white than in black populations [2]. Migraine-related pain is usually accompanied by vomiting and nausea, vaso-motor symptoms (sweating), photophobia, or phonophobia. The frequency of attacks is extremely variable, from a few episodes during a lifetime to several times a week with an average of one to three headaches a month. [3]. Chronic migraines (CM) affect 2%–3% of the general population and evolves from episodic migraines in susceptible individuals through a sequence of mechanisms including central sensitization and cortical hyperexcitability [4]. The two major types of migraine are the migraine without aura (MO) and the migraine with aura (MA). The clinical picture of MO consists of headaches with associated symptoms such as unilaterality, pulsatility of character, variable intensity, nausea, and photophobia. Routine physical activity usually aggravates migraine attacks, and phonophobia can also be associated. MA is characterized by focal neurological symptoms with variable duration, which precede or accompany a headache with migrainous qualities [5]. Regarding the pathophysiology of migraines, various sources indicated genetic predisposition or abnormal hyperexcitation from different areas of the central nervous system [6,7].

The female life cycle consists of hormonal milestones, such as menarche, menopause, pregnancy, and hormonal treatments. Menopause is characterized by the permanent cessation of the ovarian function, secondary to the depletion of ovarian follicles [8]. Previous studies reported a possible link between migraines and sex hormones variations, especially in the levels of estrogen, during the perimenopausal period and premenstrual period [9,10]. Although several studies showed controversial results [11,12], the link between menopausal transition and migraine attacks could not be dismissed. Overall, many surveys have shown a decrease of migraine frequency in late menopause [13] and a worsening of it during the menopausal transition [8,14,15]. The majority of these episodes were migraines without aura (MO). Up to 26% of the women reported increased migraine attacks frequency associated with premenstrual syndrome [16], and up to 70% noticed a menstrual association of their headaches. Usually, menstrual attacks are reported to be more painful and more non-responsive than those occurring elsewhere in the cycle [17]. All these women are usually predisposed to increased migraine frequency during the menopausal transition, which decreases in late menopause [16]. According to previous reports, menstrual migraines are more severe and disabling in comparison with nonmenstrual ones [18].

Migraines are also a leading cause of global disability [19,20], with a substantial socioeconomic impact [21]. However, despite the increased number of affected patients and decreased quality of life of these women, the World Health Organization (WHO) states that this disorder is still underdiagnosed and appropriate drugs are often inaccessible [21]. Potentially beneficial drugs for the cure of acute migraine attacks, such as oral non-steroidal anti-inflammatory drug (NSAID) or triptans, have been indicated [22], but a high number of women have a lack of response. Venlafaxine, Gabapentin, and Escitalopram also showed their efficiency in treating acute migraine attacks [23,24,25], and medicinal plants such as red clover, black cohosh supplements, and soy extracts are therapeutic alternatives when traditional drugs fail to manage the disease [26].

In recent years, Botulinum toxin (BoNT), which was first considered a lethal toxin, has represented a new perspective on the prevention and treatment of unresponsive migraine attacks, although the results of various surveys are very controversial. For clinical purposes, only BoNT-A and BoNT-B were used until now, their efficiencies being relatively similar. However, BoNT-A is the most used serotype of BoNT for clinical trials [27].

Pharmacological management of migraine possesses particular problems concerning both symptomatic and prophylactic treatment. Considering the favorable compliance and safety profile of BoNT-A, it can be used as an alternative treatment in elderly women who have not responded to other currently available drugs [28]. Currently, BoNT-A is approved by the FDA for the prophylactic treatment of migraines in Europe, USA, Australia, India, Brazil, Russia, Canada, and Korea [29]. The initial reports in women receiving BoNT-A in the field of dermatology noted that these injections also determined the alleviation of headaches. This was independent of the effect upon the muscle tone, and this observation marked the beginning of a wide range of clinical trials targetting the effects of BoNT-A on headaches and migraines when injected into superficial cranial muscles [30].

A few commercial preparations contain BoNT-A and despite their conventional unit labeling system, they have different potencies and generate different adverse effects. The side effects of BoNT-A are usually mild and infrequent, and they appear as a consequence of the mechanism of action of this neurotoxin. In addition, it can be controlled by injecting appropriate doses of BoNT-A in order to obtain therapeutic effects. Resistance to BoNT-A administration should be considered if no beneficial effects are noticed during the treatment and if muscle atrophy is not observed immediately after the injection. These aspects represent a significant clinical issue [31]. Using reduced—but potentially currative—doses of BoNT-A and prolonging the interval between two consecutive administrations could reduce the likelihood of developing drug resistance [31].

BoNT-A has gradually expanded to cover different clinical conditions and it is used nowadays in multiple fields of medicine, such as dermatology, neurology, ophthalmology, surgery or gastroenterology [32], as well as in the treatment of neuropathic diseases such as chronic migraines, back pain, myofascial pain, trigeminal neuropathy, temporomandibular joint disorders, osteoarthritis, pelvic pain, painful bladder syndrome and, more recently, in movement disorders such as camptocormia, mandibular dystonia, essential tremor, bruxism, different tics, levodopa-induced dyskinesia, and restless legs syndrome [33]. However, the use of BoNT-A is still controversial, with only a few of these potential applications being FDA approved. A synthesis of the clinical uses of BoNT-A is illustrated in Table 1.

BoNT type B (BoNT-B) has been used since 1999 for the therapy of several dysfunctions, such as spastic conditions, dystonia, hemifacial spasm, cerebral palsy, urinary dysfunction, sialorrhoea and hyperhidrosis, anal fissures, piriformis syndrome, chronic pain, and dermatologic affections. The efficiency of this serotype is mostly comparable to BoNT-A, but the side effects panel considerably differs. BoNT-B seems to generate more systemic anticholinergic adverse effects like conjunctival irritation, mucosal dryness, constipation, dysphagia, and urination difficulties [34]. Regarding the utilization of this serotype in the prevention or clinical management of migraines, no clinical evidence or community-based surveys are suggesting this aspect.

This paper is a review of the recently published data concerning the administration of BoNT-A in the prevention of chronic migraines and those related to hormonal variations, such as premenstrual and perimenopausal migraines.

## 2. Material and Methods

This study is a literature review on the efficiency of BoNT-A in chronic migraine, focusing on migraine related to hormonal variations, such as premenstrual and perimenopausal periods. This study was conducted based on previously published articles. With the ongoing interest in the field of BoNT-A mechanism of action on chronic migraine and also on the pathophysiology of this pathology in perimenopause and perimenstrual period, we aimed at addressing this topic, given the high burden that this neurological disorder possess for an increasing number of females.

While the literature offers a large array of double-blind, randomized, placebo-controlled studies on the efficaciousness of BoNT-A in migraines, it has been proven to be rather scarce in assessing the potential preventive and curative role of BoNT-A in premenstrual and perimenopausal migraines. Regarding the utility of BoNT-A in migraine attacks related to hormonal variations, the literature lacks population-based studies and the results are still ambiguous.

Our search for related articles was provided in CrossRef, PubMed, Medline, and GoogleScholar databases, using the medical subject headings (MeSH) keywords: Botulinum toxin type A, premenstrual migraine, chronic migraine, menopausal transition, neuropathic pain. The inclusion criteria of our study consisted of full-text original English written papers, double-blind, randomized, placebo-controlled, and prospective studies assessing the efficiency of BoNT-A on chronic and episodic migraine, and in hormonal variations-related attacks. A total number of 21 studies met our criteria.

## 3. Mechanism of Migraine Development

### 3.1. The Trigeminovascular System

Although many studies have referred to this topic, the origin of migraines remains controversial. This type of neuropathic pain consists of a paroxysmal disturbance, and several sources indicated that the pain arises from the activation of the trigeminovascular system [36,37].

This system is the only sensory innervation of the cerebral vessels [38], and its cell bodies are located in the trigeminal ganglion. The peripheral fibers, which are found in the ophthalmic division of the trigeminal nerve, make a synaptic connection with pain-producing large cranial vessels and dura mater [39]. This explains the distribution of pain in the ophthalmic territory, referred to a specific extracranial region, and named referred pain [40], although, sometimes patients experience pain at the back of the head, an area innervated by the greater occipital nerve. This manifestation can be explained by the convergence between trigeminal and cervical afferents in the neurons of the trigeminocervical complex, which includes the dorsal horns of C1–C2 segments of the spinal cord and one part of the trigeminal nucleus caudalis [41].

Migraines are a manifestation of both central and peripheral sensitization. The central sensitization is secondary to an altered processing sensory signal when the trigeminovascular neurons become hyperexcitable [42] and after activation, they spread the information without the need for further stimuli. Peripheral sensitization in migraines depends on the activation of peripheral nociceptors and the afferent fibers of nociceptive neurons increase the responsiveness to external stimuli [43]. Clinically, this type of sensitization associates throbbing headache aggravated during activities that increase the intracranial pressure [44].

The first order neuron located in the trigeminal ganglion receives intake from the meningeal vessels and transmits the message to the second-order neuron, located in the trigeminal nucleus caudalis. The information is finally projected to the third-order neuron in the thalamus [45]. This event is clinically expressed by an extracranial hypersensitivity.

As previously mentioned, the dura mater vessels are innervated by some small afferents, projected to the trigeminal nucleus caudalis through the trigeminal ganglion [46,47]. All these afferents seem to possess common epitopes to transient receptor potential cation channel subfamily V member 1 (TRPV1) and other neurotransmitters [48], which are activated by various pro-inflammatory factors, as tumour necrosis factor α (TNF-α) or nitric oxide [49]. At the level of the dorsal horn from the spinal cord, these afferents activate the neurons from lamina I and deep laminae [50]. Furthermore, these afferents, along with other small-afferent input, activate the second-order neurons.

The involvement of extracranial tissues in migraine development represented a subject of debate for many years. The recent findings showed that nerve fibers, which innervate cranial muscles, could be considered as functional collaterals, which pass through the sutures and are able to deliver the sensorial information from outside to inside the cranium [51,52].

### 3.2. Neurogenic Inflammation Theory

Neurogenic inflammation consists of a neurally mediated inflammatory response in the meninges, which is characterized by plasma protein extravasation, vasodilatation, and mast cell degranulation [53].

Both extracranial and intracranial small afferents have their cell bodies located in the trigeminal ganglion from the same side. After the activation of these distal terminals, a local depolarization produces the opening of voltage-gated calcium channels and the activation of soluble NSF attachment protein receptor (SNARES), which mobilize presynaptic vesicles to release their content. These terminals also release glutamate and peptides, such as calcitonin-gene-related peptide (CGRP) and substance P (SP) [54,55]. This release of peptides in the periphery produces neurogenic inflammation [29]. At the central level, this event determines the activation of second-order neurons [35]. CGRP is a protein generated by the activated trigeminal system, which produces cerebral vasodilatation and conveys nociception [56] and its release stimulates the generation of inflammatory cytokines, which play an essential role in migraine. A study by Han D. [57] showed that several interleukins such as IL-6, IL-1β, and TNF-α, were more increased in women with migraine syndrome in comparison with healthy women. Moreover, plasmatic concentration of CGRP was positively associated with IL-1β, and not related to IL-2, IL-10, and TNF-α levels.

The nociceptive information trasmitted by the meningeal vessels reaches the trigeminal caudalis nucleus through primary-afferent Aδ and C type nerve fibers arising from the trigeminal ganglion neuron [58]. CGRP produces the dilatation of meningeal vessels by increasing the blood flow [59]. Several studies reported elevated concentrations of CGRP during migraine attacks [60,61] It also mediates the histamine release from mast cells and interacts with nitric oxide (NO), a potent vasodilator, which is found in menigeal vessels [62,63]. The vasodilation that resulted may also play an essential role in the peripheral sensitization of perivascular fibers, but the exact role of these molecules during migraine attack still needs to be investigated. While CGRP possesses an obvious vasodilatory effect, SP usually increases the vascular permeability after the activation of the trigeminal system [64].

**Figure 1:**
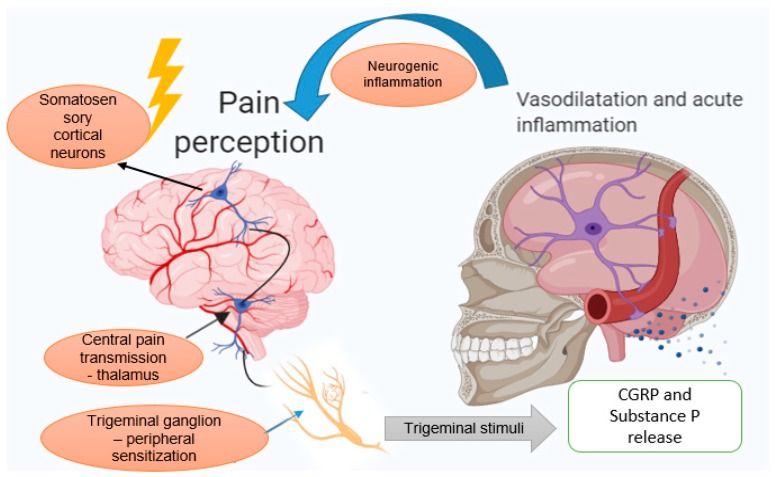
Mechanism of migraine development—migraine attacks represent a manifestation of central and peripheral sensitization. The first-order neuron in the trigeminal ganglion receives input from the dural blood vessels and the signal is transmitted to the second-order neuron in the trigeminal brainstem nuclear system and therefore to the third-order neuron in the thalamus. The sensitization of the third-order neuron in the thalamus is expressed clinically by extracranial hypersensitivity. The second accepted mechanism for migraine development consists of neurogenic inflammation, a neutrally mediated inflammatory response of the meningeal tissue characterized by vasodilatation and mast cell degranulation [42,51,67]. When the trigeminal ganglion is activated, calcitonin-gene-related peptide (CGRP) and substance P is released. These neuropeptides are considered triggers for vasodilatation, inflammation, and pain [68]. The nociceptive information is transmitted from meningeal blood vessels to the trigeminal nucleus through Aδ and C type nerve fibers arising from the trigeminal ganglion. While CGRP has a vasodilatory effect, substance P (SP) increases vascular permeability in response to trigeminal nerve activation [69].

Spreading depression is a neurophysiologic phenomenon with self-propagation, which is described as a spreading depolarization of the neural cells associated with moderate activity of the brain. This phenomenon usually underlies MA, but possibly occurs in cases without aura [65]. Recent investigations showed that spreading depression is able to induce the activation of trigeminal ganglia and to produce some changes in the vascular system, such as a hyperperfusion followed by a long period of hypoperfusion. Peripheral trigeminal stimuli generate pain and the signal is sent to the corresponding area of the somatosensory cortex through consecutive synapses [66]. Below, Figure 1 illustrates the mechanism of migraine development schematically.

## 4. Migraine Induced by Hormonal Variations

Migraine occurrence may be influenced by several hormone-related events in a woman’s life, such as menarche, menstrual cycles, menopause, pregnancy, and the use of hormonal medications, such as hormone replacement therapy (HRT) or oral contraceptives. Four of ten women before the age of 35 years contract a migraine in their lifetime [70]. Usually, the onset of migraines occurs immediately after the menarche. Then, a peak is registered during menstruation, especially in the first and second day of vaginal bleeding [71,72]. Migraines usually ameliorate during the pregnancy period and after menopause [73]. This pattern of the attacks seems to be under hormonal influence. Estrogens are able to influence the neural excitability and cerebral circulation. Furthermore, the administration of hormonal treatments may provoke a higher frequency of acute migraine attacks [73].

The “estrogen withdrawal” hypothesis is mostly considered to explain how the ovarian hormones are able to trigger the migrainous syndrome. Migraines are often produced by a sudden decline of estradiol (E2), characteristic for the premenstrual period, the menopausal transition, and the early menopause period [74]. Menopause is characterized by the loss of ovarian function, and the menopausal transition is associated with varying hormonal status. Chronic elevation of the Follicular-stimulant hormone (FSH) levels produces follicular desensitization of FSH, leading to failure of the final maturation of the ovarian follicles, resulting in anovulatory menses. The transitory enhancement of E2 level can occur as a result of the stimulation of the resting follicles by FSH. Once no more functional follicles remain, E2 also decreases, and the final cessation of menses occurs [75].

During menopause, migraines can also be provoked by the interruption of exogenous administration of estrogens. Several actions of estrogens have been described, such as the increase of vascular tone, which can explain the role of this hormone in the aggravation of migraines. E2 can also induce vasodilation through a mechanism that involves the arterial endothelium and by endothelial-independent actions. On the other hand, progesterone can induce both vascular smooth muscle relaxation and constriction [76]. According to recent findings [77], the relationship between hormonal disturbances and migraine syndrome is based on the cerebral effects of gonadal hormones (estrogen and progesterone). Thus, while E2 is able to stimulate the excitability of neurons, progesterone produces the inhibition of central neurons, increasing the susceptibility to migraines. The extreme values of plasmatic progesterone, such as excessive increases or shallow values, have been commonly linked to a higher incidence of migraine. Decreased levels of E2 induce increased production of serotonin, reduce serotonin reuptake, and degradation. During the menopausal transition and premenstrual period, several changes in serotonin concentration were described at the trigeminal ganglion level [77]. The consequent cyclical changes in serotonin levels in trigeminal ganglia could contribute to the selective response to estrogen withdrawal. Given that serotonin has also been involved in the pathophysiology of vasomotor symptoms, a common pathophysiology could be considered.

A drop of estrogen levels during menstration and menopausal transition decreases the serotonin level and can bring on a migraine [10]. In fact, the variations of E2 levels are the main trigger for migraines, rather than the reduced plasmatic values of it. This hormone is able to regulate the neuroactive molecules. It has been discovered that estrogen rapidly increases the NO in cerebral circulation by increasing the level of nitric oxide synthetase (NOS) through the PI3K/Akt/eNOS pathway [78]. Estrogen receptors (ER) ERα and ERβ are expressed in the trigeminal ganglion and within dorsal raphe, respectively [79]. After estrogen binds to its receptors, the complex ligand-receptor enters the nucleus, binds to DNA regulatory regions [80], and regulates the levels of CGRP, serotonin, and NO.

The serotonin receptor system includes several receptor types, from which 5-HT1 has been identified as being the most responsible for migraine activity. This could be explained by the presence of serotonin receptors in the trigeminal nerve endings, and the involvement of 5-HT3 receptors in acetylcholine release [81].

Therefore, estrogen variations are highly related to the rise or aggravation of migraines by altering the pain sensitivity, the excitability of the neuronal cells, and the brain vasoactivity [82].

## 5. Botulinum Toxin

BoNTs are secreted by a bacteria of the genus *Clostridium*. *Clostridium botulinum* is the most widespread bacteria and also the most studied of this genus. Four groups of *Clostridium* are described. This bacteria produces seven serotypes of BoNTs, marked from A to G, and more than 40 subtypes [83,84,85,86].

Although all BoNT serotypes share the same structure and function, BoNT-A and BoNT-B are the most used for clinical practices. BoNT-A is a double-chain protein, which was first identified in 1919 by Georgina Burke [86] and weighs approximately 900 kDa. This neurotoxin is composed of a light chain (LC) and a heavy chain (HC) of approximately 50 kDa and 100 kDa, respectively, linked by a disulfide bond [87]. The HC binds BoNT-A to presynaptic gangliosides on the cellular membrane and promotes the translocation of LC across the endosomal membrane [88,89]. The LC of BoNT-A, BoNT-C, and BoNT-E, which is an endopeptidase, cleaves the SNARE, a membrane protein [87]. Three SNARE proteins were described, and the segregation of any one of them blocks the interference between synaptic vesicles and plasmatic membranes, preventing the release of neurotransmitters from neuronal cells [90,91].

BoNTs are able to inhibit the exocytosis of Acetylcholine (ACh), and this property has been used in clinical practice in order to treat a wide range of muscular disorders [92]. The process of ACh exocytosis requires the SNARE proteins in the presynaptic membrane: Syntaxin, synaptosomal-associated protein-25 kDa (SNAP-25), and VAMP/synaptobrevin. These proteins are cleaved by the LC into the cells. This cleavage interferes with SNARE-mediated protein transport and transmitter release, blocks muscle innervation at the neuromuscular junction, and results in a temporary paralysis [93,94].

BoNT-A and BoNT-B have proved their efficiency in the therapy of neuropathic pain, but BoNT-A is more widely used due to its less pronounced adverse effects and its long-lasting results [95,96]. Current findings based on clinical surveys support the therapeutic and preventive potential of BoNT-A in chronic migraines. Regarding migraine attacks related to hormonal variations, such as menstrual or perimenopausal migraines, unresponsive to other treatments, the New York Headache Center indicates BoNT-A as the best preventive therapy [97], but further clinical trials are necessary to support this.

## 6. BoNT-A—Mechanisms of Action in Chronic Migraine

The mechanisms of action of BoNT-A in chronic migraines are very complex, and several hypotheses have been suggested. Following the local stimulation, the peripheral terminals of sensorial afferents mediate the release of CGRP and SP [98], leading to mast cell degranulation and plasma extravasation, which activates the peripheral nerve terminals [49]. BoNT-A decreases the neurogenic inflammation, inactivates Na_+_ channels, and exhibits the transport through axons [99].

The next step is the mobilization of SNARE proteins through the plasmatic membrane, leading to the release of ACh, SP, and CGRP, which induces protein extravasation. SP is contained in primary-afferent neurons and activates the NK1 receptor located in dorsal horn neurons. Activated NK1 receptors are internalized into the dorsal horn, and this phenomenon could be reversed by the administration of BoNT-A by reducing SP release. The role of NK1 receptors is to reduce the response to pain. They can also reduce the activation of c-Fos, which is known as a marker for the activation of nociceptive neurons, in different areas of the brain [69,100,101].

The release of neurotransmitters and the consequences of this action were imitated by the local delivery of the TRPV1 channel agonist capsaicin [102]. This phenomenon was blocked by the peripheral administration of BoNT-A. Durham et al. [103] utilized cultures of the trigeminal system from rats, stimulated the trigeminal system with capsaicin, and treated the cultures with BoNT-A. The results indicated that BoNT-A possesses the ability to suppress the release of CGPR from activated sensorial neurons.

BoNT-A can diminish the pain by preventing the fusion between the plasmatic membrane and presynaptic vesicles. The injection of BoNT-A at peripheral sites affects the discharge of neurotransmitters into the presynaptic areas, in postsynaptic neurons [104], and also interrupts the transfer of various receptors through the synaptic membranes. These receptors are TRPV1 and transient receptor potential ankyrin 1 (TRPA1) [51]. TRPA1 is a polymodal ion channel expressed in a subset of nociceptive neurons of the trigeminal ganglion and dorsal root ganglion (DRG), and its activation produces pain and neurogenic inflammation [105]. TRPV1 is the most well-known member of the TRP- family. It is a ligand-gated cation channel able to respond to various stimuli, which possesses an essential role in pain modulation, vasculature tonus, and neurogenic inflammation. Up-regulation of TRPV1 expression in the DRG represents one of the mechanisms of neuropathic pain. The inhibition of hyperalgesia by BoNT-A is most likely mediated through the reduction of TRPV1 expression in the nociceptors [106].

While the peripheral action of BoNT is widely recognized, the central mechanism of action is yet to be discovered. Because BoNT-A is a large molecule, it is almost impossible for it to pass the blood–brain barrier. Two methods through which BoNT-A reaches the central nervous system (CNS) were described: Systemic spread and axonal transport (both anterograde and retrograde) [107]. The hypothesis of passive diffusion of BoNT-A was also suggested [108], but the axonal transport is the most plausible mechanism that could explain the transport of BoNT-A in both, various regions of nerve endings, and CNS [109]. Restani and coworkers [110] delivered BoNT-A into the eyes of rats and showed a significant SNAP-25 cleavage in tectum, produced by BoNT-A. The results of the study confirmed the theory of anterograde transport and transcytosis of BoNT-A in axons, instead of passive diffusion. Furthermore, Antonucci et al. [111] analyzed the facial motor nucleus after the injection of BoNT-A and observed a deep division of SNAP-25. This event highlighted the possibility that BoNT-A is retrograde transported and transcytosed to central neurons and motor neurons. In addition, bilateral effects have been noticed after the administration of BoNT-A unilaterally [112].

Remarkably, BoNT-A is believed to act only on chronic or hypersensitive pain, not on acute or imminent pain. This lack of effect on acute pain perception shows that the antinociceptive action of BoNT-A is a simple block of the afferent terminal release [113].

The axonal organelles, which travel through the microtubules, regulate the transport of BoNT-A along the axons to the DRG and dorsal horn. Then, the toxin spreads to nearby glial and neuronal cells [114]. To date, a wide range of studies suggested that BoNT-A produces a reduction in peripheral sensitization immediately after the injection. The next step is the indirect reduction of the central sensitization [115,116,117]. In migraines, BoNT-A specifically targets areas of the CNS after the strategic peripheral injections into anatomically connected sites. Using an experimental animal model, researchers have demonstrated a strong link between the nociceptive cervical and meningeal afferent nerve fibers, such as the coupling between the dura and the greater occipital nerve [118].

According to the phase III research evaluating migraine prophylaxis therapy protocol (PREEMPT), BoNT-A administration into the occipital muscles, which are located near the occipital nerve fibers, could influence the nociception of the meninges due to axonal transport. Using the same mechanism of retrograde transport through the axons, BoNT-A injections into the trigeminal nerve terminals may affect the meningeal afferent tracts on the trigeminal complex [119].

A recent study by Schueler et al. [52] described a new network of fibers, which crosses the cranial sutures and goes from the meninges to extra-cranial tissues. This observation raised the possibility that the injection site of BoNT-A targets the suture lines, in contrast to PREEMPT protocol, which targets pericranial muscles.

**Figure 2:**
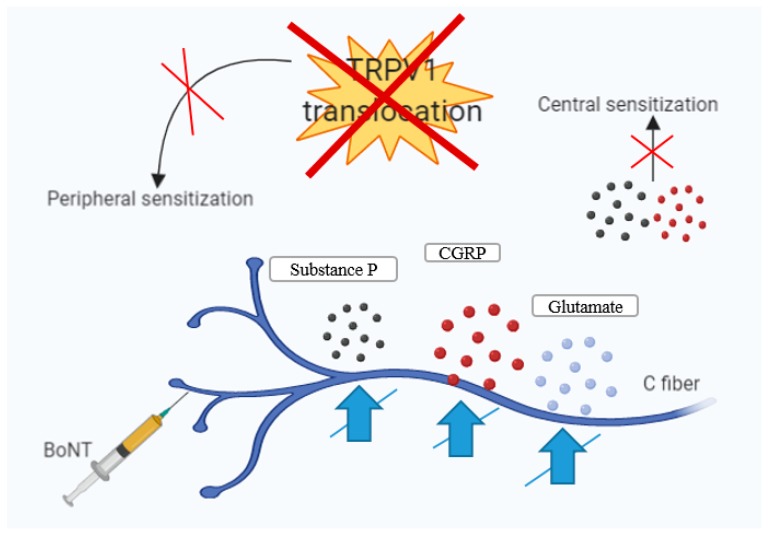
BoNT-A mechanism in migraine—botulinum toxin enters the nerve and inhibits the neurotransmitters, both pro-inflammatory and nociceptive, leading to an attenuation of inflammation, vasodilatation, and finally, of pain. Moreover, the modulation of this event contributes to the decrease in pain intensity. Furthermore, BoNT-A produces local desensitization, and the toxin decreases plasmatic concentrations of SP, glutamate, and CGRP, which are the triggers for pain, inflammation, and vasodilatation. BoNT-A interferes with the transport through the axons to neighboring areas, and the same effects was observed in adjacent neurons or glial cells [104,120,121].

Figure 2 illustrates the BoNT-A mechanism of action in migraine. It inhibits the release of neuropeptides and glutamate, leading to a peripheral desensitization and, indirectly, to central desensitization.

## 7. Results and Discussion—Clinical Studies of BoNT-A on Migraine

Systematic clinical trials using BoNT-A as a prophylactic treatment for migraine resulted in a mixture of positive and negative findings. During our research, we found no clinical trials based strictly on the effects of BoNT-A in menstrual or perimenopausal migraine prophylaxis.

BoNT-A (Botox^®^) was approved by the FDA for the prophylactic treatment of CM in 2010 [81], but the injection location is still controversial. Currently, the FDA has approved an administration pattern for BoNT-A, which includes the chemodenervation of several muscular groups from the level of the neck and head, which are as follows: Corrugators, frontalis, occipitalis, temporalis, procerus, cervical paraspinal, and trapezius. BOTOX^®^ is injected bilaterally at the level of these muscles in doses of 10U, 20U, 30U, 40U, 5U, and 20U, respectively. However, in the literature, another injection pattern has also been described: The technique consists of the administration of the toxin directly into the peripheral nerves. Some anatomical areas are targetted and a smaller quantity of BOTOX is administered. The locations for the direct administration of BoNT-A into the nerves include some peripheral nerve trigger areas that are described as contributors to the pathogenesis of CM [122].

In Europe and the USA, there are three different brands of BoNT-A used in the clinical practice: Anabotulinum toxin A, ona botulinum toxin A, and incobotulinum toxin A. All these products have an identical active compound, a 50 kDa LC, and their efficiency is comparable when used in equivalent dosages [123]. Onabotulinum A (Botox^®^, Allergan) is a medical drug with a molecular weight of about 900 kDa and is one of the most used BoNTs for medical and esthetic indications, including the prophylaxis of CM.

The results of all the reviewed studies indicate that BoNT-A is both useful and safe as prophylactic agent for CM, but it is not effective in episodic migraines (EM). EM represents a migraine attack that does not meet the established criteria for CM, and is characterized by less than 15 headache days per month [124]. Meanwhile, CM is defined as repetitive headache attacks, which appear for at least 15 days/month during a period of three months [125]. In midlife, studies suggested that the incidence of migraines usually increases during the menopausal transition. Also, it appears more often in women who developed perimenstrual migraines [126]. Analyzing the results of BoNT-A administration for the prophylaxis of CM and EM, we could open a new therapeutic perspective for the management of menstrual and perimenopausal migraine, but further surveys are imperative.

Onabotulinum A (Botox^®^, Allergan) is the only commercial product containing BoNT-A, approved for the treatment of CM, while incobotulinumtoxin A and abobotulinumtoxin A have controversial and not well-documented results. Kazerooni and collegues [127] analyzed a retrospective case series including 21 patients with CM, which were treated with incobotulinumtoxin A. The results indicated major improvements in the severity and frequency of migraine attacks, but further investigations on human models should aim to clarify its efficiency in migraines. Abobotulinumtoxin A (Dysport^®^, Beaufour Ipsen, Boulogne-Billancourt, France) was poorly investigated in women with EM, and the results indicated statistically insignificant results on the prevalence of these episodes. Furthermore, no evidence for the usefulness of Abobotulinumtoxin A for patients with CM was recorded.

Various doses and administration sites of BoNT-A have been addressed over time. Phase III research evaluating migraine prophylaxis therapy (PREEMPT) study group launched two hypotheses regarding the injection location. For one side, they mentioned 31 fixed sites for administration, consisting of the muscles frontalis, corrugators, procerus, occipitalis, temporalis, trapezius, and cervical paraspinal group. The alternative consisted of the sites following the pain. Usually, the concentration of onabotulinumtoxinA should be of 5 MU/0.1 mL, and it is obtained after the dilution of 50 MU of neurotoxin with 2 mL of saline solution. The PREEMPT trials used single injections of BoNT-A (BOTOX ^®^) every 12 weeks and the investigators proved the efficiency of this agent. For BoNT-A, the relapse period occurs approximately within four months, and could reflect the time required for the return of the frequency of headache days back to baseline.

It was demonstrated that BoNT-A can reduce the pain induced by capsaicin in the trigeminal system. In 2009, Gazerani et al. [128] conducted a study in humans pre-treated with subcutaneous BoNT-A, administered unilaterally on the brow, after the induction of pain with capsaicin. On the opposite side, they administered a saline solution. After the administration of the BoNT-A injection, the intensity and area of pain, the area of secondary hyperalgesia, and the vasomotor-associated symptoms were analyzed by the researchers. They observed that BoNT-A reduced the intensity of the induced pain, the secondary hyperalgesia, and the associated vasomotor symptoms such as flare and increased skin temperature, suggesting that BoNT-A primarily targets C- fibers.

Kollewe and colleagues [129] performed a prospective study on 27 patients suffering from CM (25 females and two males aged 56 ± 10.8 years) in order to analyze the performance of onabotulinumtoxinA (Botox(^®^)) in chronic usage. They injected the substance into the locations mentioned by PREEMPT studies, and the period of administration was 73 ± 37 weeks. After this period, 96% of the cases reported improvements in their general status. The number of headaches days per month was significantly reduced, as well as the total number of days with migraines. In addition, the number of autonomic days increased, and the days with analgesic medication were significantly reduced. The quality of life improved in 96% of cases, and adverse effects were transient and mild. The authors concluded that BoNT-A is highly useful in treating CM and safe for chronic administration.

The peak of onabotulinumtoxin A efficiency and safety was highlighted when the PREEMPT study group made the results of their two clinical trials public: PREEMPT I and PREEMPT II. The two placebo-controlled studies enlisted 1384 cases and consisted of a 28-day baseline screening period, a 24-week double-blind, parallel-group, a placebo-controlled phase (two injection cycles), and a 32-week open-label phase (three injection cycles). The primary endpoint of the PREEMPT I trial, consisting of the reduction of migraine episodes, was missed, although significant differences between the two groups were recorded [130].

In 2010, Diener and coworkers [131] published the results of the PREEMPT II clinical trial. In comparison with the study design of PREEMPT I, this time, the cohort was randomized into two groups, as follows: The first cohort included 347 patients and received between 155 U and 195 U of BoNT-A, and the placebo group included 358 subjects. The treatment was administered during 12 weeks in two cycles. From the beginning to weeks 21–24, onabotulinumtoxinA confirmed its efficacy in considerably decreasing the number of pain days in comparison to the placebo.

Topiramate was the first line drug used for the prevention of CM in association with the removal of the risk factors [132]. In their multicentric pilot study, Cady et al. [133] revealed that Onabotulinumtoxin A and topiramate possess the same yield for patients with CM. They randomly assigned 59 subjects into two groups (30 and 29 cases), which received topiramate tablets associated with placebo injections and onabotulinumtoxinA injections associated with oral placebo. The patients self-reported their symptoms in diaries during 12 weeks of the follow-up period. The primary endpoint followed the response rate and showed improvements in both groups of study. The secondary endpoints consisted of the number of headache days per month, the total number of days with acute attacks, pain-free days, analgesic medication ingestion, and the severity degree of the attack. After the follow-up period, the authors obtained a similar efficiency of OnabotulinumtoxinA and topiramate for adults with CM.

The first evidence for the positive outcome of BoNT-A was described in subjects who were undergoing this treatment for the therapy of facial wrinkles. The first non-randomized study included 106 patients, of which 77 were diagnosed with CM. These subjects were injected with onabotulinumtoxinA (Botox^®^, Allergan Inc., Irvine, CA, USA), and the benefits were subjectively appreciated through their self-reports. Of the 77 treated subjects, 51% and 28%, respectively, reported a complete and a partial result in migraine alleviation [134].

The first placebo-controlled trial realized on CM patients was performed by Silberstein et al. [135] and included 123 patients with MA and MO. The patients were divided into two groups who received only one injection of a vehicle or 25 U/75 U of BoNT-A (BOTOX; Allergan, Inc). The toxin was injected into the pericranial muscles during the first visit. After the administration, the follow-up spread over a three-month period. During this time, the subjects kept track and evaluated the incidence and severity of the attacks, as well as the occurrence of vasomotor symptoms. The study concluded that pericranial injection of 25U of BoNT-A represents a safe treatment, which can reduce the severity and frequency of acute headaches, analgesic drugs ingestion, and migraine-associated vasomotor symptoms.

Mathew and colleagues [136] realized a placebo-controlled, randomized, and double-blind study, in order to assess the tolerability and utility of BoNT-A for the prophylaxis of CM. They included 571 subjects aged between 18 and 65 years old, which experienced at least 16 episodes of headaches during a month. The patients received either BoNT-A (BOTOX^®^, Allergan, Inc.) or placebo, daily for 90 days. The primary endpoint consisted of an analysis of the attacks’ frequency and headache-free days per month. The secondary endpoint consisted of the decline of headache frequency with at least 50% compared to baseline. Of the 571 cases initially considered for this study, only 355 were finally included and randomized. At the end of the placebo run-in period, 279 subjects were considered placebo non-responders, and 76 were classified as placebo responders. Then, the subjects were randomized and received placebo or BoNT-A injections. The investigators concluded that the differences between the two groups after the first end-point time were statistically insignificant. In addition, the severe decline of headache frequency during the follow-up period was statistically significant. Regarding the safety profile of BoNT-A, it presented satisfying tolerability in subjects with chronic headache. In a few cases, treatment-related side effects were inconstant and mild. The side effects of this therapy are usually local and related to the injection location. Moreover, the systemic side effects are uncommon. Among the reported adverse effects, the most common are muscular weakness (1.6%), chronic cervical pain (4.3%), local pain at the injection area (2.1%), and ptosis of the eyelid (1.9%), but this treatment is usually safe and tolerable [130].

Hou et al. [137] created a new perspective of the treatment with Onabotulinumtoxin A in the therapy of migraines. The authors combined the positive effect of acupuncture with the efficiency of BoNT-A. The administration of Onabotulinumtoxin A in small doses into some specific acupoints represents an enigmatic branch of the classic acupuncture from the traditional Chinese medicine. The authors conducted a prospective study, which included patients with both EM and CM and also a control cohort of subjects. BoNT-A was injected in predetermined fixed locations, including the muscles temporalis, occipitalis, frontalis, corrugator, and trapezius or at acupoint-sites, known as Fengchi (GB20), Taiyang (EX-HN5), Tianzhu (BL10), Baihui (GV20), Shuaigu (GB8), and Yintang (EX-HN3). After a follow-up period, the authors concluded that BoNT-A injected either in predetermined sites, or in some specific points used in acupuncture, was able to reduce significantly the intensity, frequency, and time of migraine acute attacks. Also, the vasomotor symptoms associated with migraines were ameliorated in the study group. Comparing the efficiency of BoNT-A depending on the injection site, the results indicated that the injection into specific acupoints showed more usefulnesss compared with fixed-places injections.

Therapy studies of BoNT-A on CM indicated that its efficacy increased over time, up to 56 weeks, concomitant with a greater improvement of the quality of life [138]. One study also suggested that the effectiveness of BoNT-A was associated with headache perception and that imploding migraine headache is more likely to be prevented by BoNT-A, rather than the exploding headache [139].

Out of 11 clinical trials performed with EM patients, only two showed a positive effect of BoNT-A in reducing the frequency of attacks in comparison with placebo. A meta-analysis performed on these studies concluded that BoNT-A is efficient only for CM and had no effect on EM [140]. In contrast, BoNT-A was associated with a likelihood of 50% or greater improvement for the patients suffering from CM [140].

All the studies included in our research had a number of subjects that did not respond to BoNT-A therapy. More than 10% of these cases are considered non-responders because of the long period of chronic administration. Another reason invoked for the development of treatment resistance consisted in the apparition of antibodies. This phenomenon is considered an initial placebo effect or an intrinsic worsening of the migrainous syndrome [141].

In Table 2, based on the trials thus far reported, we summarize the effects of BoNT-A in CM and EM therapy and prevention. 

## 8. Conclusions and Future Perspectives

Based on the results of past studies, BoNT-A (BOTOX^®^) is a promising agent for the prophylaxis of migraines. Despite its frequent utilization and the multitude of related studies, there are no conclusive data regarding the pathways and central effects of this neurotoxin.

BoNT-A is most widely used for the prophylaxis of migraines, and there is a growing amount of evidence that this therapy leads to a significant decline of headache attacks per month. Also, the quality of life of the affected patients significantly improved after the administration of this neurotoxin. BoNT-A doses ranged between 150 U and 195 U and were efficacious with limited side effects. Usually, the time of onset of the therapeutic effect on migraine was after three months of administration. A meaningful reduction in headache was observed at week 56. No clinical trials investigated the efficacy of BoNT-A in migraines related to hormonal variations.

In conclusion, further studies are still required in order to thoroughly understand the mechanism of action of BoNT-A in migraines, its central actions, and analgesic properties. A setup of better designed surveys could improve the identification of BoNT-A responders and allow a more tailored prophylactic treatment for migraines. This would allow broadening the scope of clinical applications of this product and its use in new medical conditions, such as prevention and treatment of migraines related to hormonal variations (menstrual and perimenopausal migraines).

Our study also paves the way for future clinical trials on this topic.

## Figures and Tables

**Table 1:** Clinical uses of Botulinum toxin A (BoNT-A) and Botulinum toxin B (BoNT-B) in medicine [32,35].

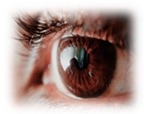	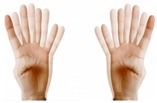	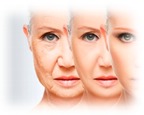	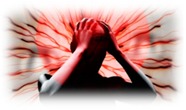	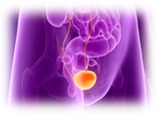	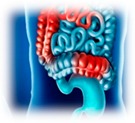	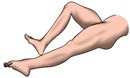
Ophthalmic Disorders	Movement Disorders	Cosmetic Applications	Chronic Pain	Genito-Urinary System Disorders	Gastrointestinal Disorders	Other Conditions
Established Indications Of Bont (Approved by FDA)			Tried Applications of Bont			
Strabismus	Cervical dystonia	Wrinkles	Migraine	Chronic pelvic pain	Achalasia	Cerebral palsy
Concomitant misalignment	Oromandibular dystonia	Axillary Hyperhidrosis	Tension type headache	Vulvodynia	Bruxism	Spinal cord injury
Restrictive or myogenic strabismus	Torticollis	Lateral canthal lines	Lower back ache	Detrusor-sphincter dyssynergia	Palatal myoclonus	Various brain injuries after trauma
Eyelid retraction	Tardive dystonia	Glabellar lines	Myofascial pain	Spasms of perineal musculature	Chronic anal fissures	Upper-limb spasticity
Duane’s syndrome	Other focal dystonias	Browlift	Tennis elbow	Dyspareunia	Larynx affections	Hemifacial spasms
Non-concomitant Misalignment			Trigeminal neuropathy	Painful bladder syndrome	Temporomandibular joint dysfunction	Stoke-induced spasticity

**Table 2:** Clinical studies on BoNT-A efficiency in migraines.

Author, Reference	Study Design	No. of Patients	Type of Migraine	Dose of BoNT-A	Results	Follow-Up Period
Saper et al., 2007 [142]	Randomized, Double-Blind, placebo-control study	232 patients (45 placebo)	EM	25 U BoNT-A	Both BoNT-A and placebo had similar efficiency, and showed greater reduction of migraine severity	3 months
Evers et al., 2004 [143]	Randomized, Double-Blind, placebo-control study	60 patients (20 placebo)	EM	16 U or 100 U BoNT-A	Both BoNT-A and placebo decreased the number of migraine days and the frequency of the attacks	3 months
Vo et al., 2007 [144]	Randomized, Double-Blind, placebo-control study	32 patients (17 placebo)	EM	205 U BoNT-A	No significant reduction of migraine frequency and severity was registered The headache pattern index indicated a protective effect for BoNT-A against attacks severity	3 months
Petri et al., 2009 [145]	Randomized, Double-Blind, placebo-control study	127 patients (63 placebo)	EM	80–120 U BoNT-A into cervical and pericranial muscles	BoNT-A was not useful as a prophylactic treatment; the reduction of headache did not reach statistically significance	3 months
Chankrachang et al., 2011 [146]	Randomized, Double-Blind, placebo-control study	128 patients (37 placebo)	EM	120–240 U BoNT-A	BoNT-A was significantly useful over placebo	8–12 weeks
Anand et al., 2006 [147]	Randomized, Double-Blind, placebo-control study	32 patients (16 placebo)	EM	50 U BoNT-A	75% of patients reported a complete relief of the symptoms	3 months
Relja et al., 2007 [148]	Randomized, Double-Blind, placebo-control study	515 patients	EM	75–225 U BoNT-A	Similar results in both groups	9 months
Silberstein et al., 2000 [135]	Randomized, Double-Blind, placebo-control study	123 (41 placebo)	EM	25 U or 75 U BoNT-A	Greater results in both groups	3 months
Elkind et al., 2006 [149]	Randomized, Double-Blind, placebo-control study	182 patients (100 placebo)	EM	7.5 U–50 U BoNT-A	No improvements in headache were noted, no differences between BoNT-A and placebo	120 days
Barrientos and Chana, 2003 [150]	Randomized, Double-Blind, placebo-control study	30 patients (15 placebo)	EM	50 U BoNT-A	The number of attacks per day and headache frequencies were significantly reduced on day 90	3 months
Freitag et al., 2008 [151]	Randomized, Double-Blind, placebo-control study	86 patients	CM	100 U BoNT-A	BoNT-A was superior to placebo for both endpoints	4 months
Cady et al., 2011 [133]	Randomized, Double-Blind, placebo-control study	59 patients (30 topiramate)	CM	300 U BoNT-A	Similar results for both BoNT-A and Topiramate	26 weeks
Diener et al., 2010 [131]	Randomized, Double-Blind, placebo-control study	679 patients (338 placebo)	CM	155–195 U BoNT-A	All the secondary endpoints were favoured	32 weeks
Binder et al., 2000 [134]	Non-randomized, open-label	106 patients	CM		−51% of cases—complete response −38% of cases—partial response −70% of cases—improvements were observed after one hour of injection	3 months
Magalhaes et al., 2010 [152]	Randomized, Double-Blind, placebo-control study	72 patients (23 amytriptiline)	CM	250 U BoNT-A	No difference was observed between BoNT-A and amytriptiline effects	90 days
Mathew et al., 2009 [153]	Randomized, Double-Blind, placebo-control study	60 patients (29 topiramate)	CM	200 U BoNT-A	Similar results for both groups. BoNT-A and Topiramate showed similar efficiency	9 months
Aurora et al., 2011 [154]	Randomized, Double-Blind, placebo-control study	1384 patients (696 placebo)	CM	155–195 U BoNT-A	BoNT-A was efficient in improvement of the total headache days number	56 weeks
Aurora et al., 2010 [130]	Randomized, Double-Blind, placebo-control study	679 patients (338 placebo)	CM	155–195 U BoNT-A	BoNT-A was efficient in improvement of the headache days number but no reduction in the migraine episodes was recorded	24 weeks
Lipton et al., 2011 [138]	Randomized, Double-Blind, placebo-control study	1384 patients (696 placebo)	CM	155 U BoNT-A	Significantly reduction in headache compared to placebo	56 weeks
Mathew et al., 2005 [136]	Prospective Study	571 patients	CM	105–260 U	50% or more decrease in the frequency of headache days was registered at 180 days	11 months
Dodick et al., 2009 [119]	Randomized, placebo-control study	1384 patients	CM	155–195 U	BoNT-A considerable decreased the number of pain days in comparison to placebo.	24 weeks

## Abbreviations

*ACH*	Acetylcholine
*BONT*	Botulinum toxin
*BONT-A*	Botulinum toxin serotype A
*BONT-B*	Botulinum toxin serotype B
*CGRP*	Calcitonin gene-related peptide
*CM*	Chronic migraine
*CNS*	Central nervous system
*DA*	Dalton
*E2*	Estradiol
*EM*	Episodic migraine
*FDA*	Food and Drug Administration
*FHM*	Familial hemiplegic migraine
*FSH*	Follicular-stimulant hormone
*HC*	Heavy chain
*IL*	Interleukin
*LC*	Light chain
*MA*	Migraine with aura
*MIDAS*	Migraine Disability Assessment
*MO*	Migraine without aura
*MU*	Mouse Units
*NO*	Nitric Oxide
*NOS*	Nitric oxide synthase
*NSAID*	Non-steroidal anti-inflammatory drug
*PREEMPT*	Phase III Research Evaluating Migraine Prophylaxis Therapy
*QOL*	Quality of Life
*SNAP-25*	Synaptosomal-associated protein-25 kDa
*SNARE*	Soluble NSF attachment protein receptor
*SP*	Substance P
*TNF-* *A*	Tumor necrosis factor alpha
*TRP*	Transient receptor potential channel
*TRPA1*	Transient receptor potential ankyrin 1
*TRVP1*	Transient receptor potential cation channel subfamily V member 1
*VAMP*	Vesicle-associated membrane protein
*WHO*	World Health Organization
